# Bioactive Compounds Derived from Plants and Their Medicinal Potential

**DOI:** 10.3390/ph18111732

**Published:** 2025-11-14

**Authors:** Sónia M. R. Oliveira, Ana Paula Girol, Veeranoot Nissapatorn, Maria de Lourdes Pereira

**Affiliations:** 1 CICECO-Aveiro Institute of Materials, University of Aveiro, 3810-193 Aveiro, Portugal; 2 Experimental and Clinical Research Center (CEPEC), Padre Albino University Center (UNIFIPA), Catanduva 15809-144, SP, Brazil; 3 Futuristic Science Research Center, School of Science, World Union for Herbal Drug Discovery and Research Excellence Center for Innovation and Health Products (RECIHP), Walailak University, Nakhon Si Thammarat 80160, Thailand; 4 Department of Medical Sciences, University of Aveiro, 3810-193 Aveiro, Portugal

The inaugural edition of the Special Issue “Bioactive compounds derived from plants and their medicinal potential” offers a comprehensive exploration of the therapeutic promise inherent in plant-based compounds. This collection of 17 studies highlights the diverse bioactivities of medicinal plants, including antimicrobial, anti-inflammatory, anti-cancer, and immunomodulatory effects, and their potential to address current therapeutic challenges. As previously established, plants are a source of bioactive compounds that play vital roles in pharmacotherapy, and serve as a foundation for emerging therapeutics and nutraceutical agents [1,2]. Together, these contributions employ a range of research methodologies, from in vitro assays to in vivo models, reflecting the dynamic and multidisciplinary nature of natural products pharmacology.

While technological advancements, including data science applications, are refining the study of plant-derived bioactive compounds, significant challenges remain. Among these is the difficulty of translating preclinical findings into clinical applications—the “bench-to-bedside” gap. Inconsistent efficacy and safety profiles observed in clinical trials often arise from the inherent complexity of plant extracts, variability in composition, and quality control issues.

This Special Issue addresses these gaps by presenting studies that not only examine the bioactivity of plant-derived compounds, but also investigate their mechanisms of action, explore synergistic effects, and evaluate opportunities for formulation and delivery improvements.

For example, Mateusz Kciuk et al. (2024) [3] provide a narrative synthesis on afzelin (kaempferol-3-O-rhamnoside), a flavonoid glycoside, highlighting its diverse bioactivities. Their review emphasizes afzelin’s neuroprotective effects in neurodegenerative diseases and its anti-cancer properties, including the induction of apoptosis and inhibition of cancer cell proliferation. A systematic review by Syed Mohammed Basheeruddin Asdaq et al. (2024) [4] comprehensively analyzes in vitro and clinical studies on date palm extracts (*Phoenix dactylifera*), detailing their antioxidant, anti-inflammatory, and neurogenic effects. This work positions date palm extracts as promising natural therapies for neurodegenerative conditions.

Our Special Issue also includes novel research.

Vinícius Lopes Lessa et al. (2024) [5] investigated the citrus flavonoid naringenin using in vitro promastigote assays, fractional inhibitory concentrations, and isobologram in combination with miltefosine against *Leishmania amazonensis* (a cutaneous leishmaniasis pathogen), reporting advantages of this combination strategy. The combinatorial effect of naringenin and miltefosine against *Leishmania amazonensis* exemplifies the potential of integrating natural compounds into existing pharmaceutical drugs to enhance therapeutic efficacy.

Similarly, Fekandine V. Douti et al. (2025) [6] explored the effects of spices such as *Aframomum melegueta* and *Xylopia aethiopica* on taeniasis/cestoda. Through docking analyses of known phytochemicals—such as pungent phenolics and terpenoids—they identified promising interactions with helminth targets. Although in vivo validation and constituent isolation are needed, this study highlights the need for alternative treatments to combat parasitic infections.

In an in vivo study conducted by Meng-Ting Zeng (2024) [7], ethanolic extracts from the leaves and branches of several Cupressaceae conifer species (e.g., *Chamaecyparis lawsoniana*, *Cupressus sempervirens*, and *Platycladus orientalis*), demonstrated systemic protection. The extracts rapidly prevented arrhythmias in a barium chloride (BaCl_2_)-induced arrhythmia model, an effect mediated through M2/M3 muscarinic receptors. This work represents the first in vivo demonstration of this rapid protective mechanism, elucidated with muscarinic antagonists.

Yasmen F. Mahran et al. (2024) [8] evaluated thymol (from *Thymus* spp.) in a rat model of 5-FU-induced liver injury (IP 150 mg/kg). Administering oral thymol at 60/120 mg/kg and using in silico analysis for pathway identification, the study revealed that thymol effectively reduces oxidative stress and apoptosis while modulating Akt/GSK-3beta and ERK1/2 pathways. These findings support thymol’s potential as a therapeutic agent for chemotherapy-induced hepatotoxicity.

Additionally, Jun Yang et al. (2024) [9] investigated crocetin, a saffron carotenoid, in an animal model of high-altitude hypoxia. Their results showed that crocetin mitigates oxidative stress and inflammation, protecting organs from hypoxia-induced damage.

The scope of research extends to cancer and toxicity models as well.

In an Ehrlich tumor-bearing mouse model (ascitic and solid), Lucas Sylvestre Silva et al. (2025) [10] demonstrated that leaf extracts of *Garcinia brasiliensis* inhibited tumor growth and improved survival, suggesting antitumor bioactivity.

Targeting pain and inflammation, other authors used classic rodent models to investigate crude extracts of other valuable plant species.

Gabriela Castañeda-Corral et al. (2024) [11] demonstrated that an acetonic extract of *Bougainvillea x buttiana* has potent anti-nociceptive and anti-inflammatory effects, supporting its traditional medicinal use.

Caren Naomi Aguero Ito et al. (2024) [12] further demonstrated significant analgesic and anti-arthritic activities of methanolic extract and palmatine, an alkaloid derived from *Annona squamosa* leaves, substantiating their potential in pain and inflammation management.

Finally, Salud Pérez-Gutiérrez et al. (2025) [13] identified two labdane enantiomers from *Gymnosperma glutinosum* with anti-inflammatory activity through comprehensive in vivo, in vitro, and in silico studies. This research provides insights into these active enantiomers’ mechanisms and potential therapeutic applications.

Infectious disease applications were also well represented.

Reem Binsuwaidan et al. (2024) [14] developed a lycopene–selenium nano-formulation exhibiting potent antibacterial, antioxidant, and anti-inflammatory properties. Characterized by FTIR, zeta potential, and particle size (~126 nm), the nanoparticles showed significant in vitro antioxidant and antibacterial effects and enhanced healing in a rat wound infection model. These findings suggest the formulation’s promise as a multifunctional therapeutic agent against infection and inflammation.

Looking ahead, advancing plant-based therapeutics will require bridging the gap between laboratory research and clinical applications. This will require rigorous clinical trials, improved standardization of extraction and formulation methods, and advances in pharmacokinetics and pharmacodynamics modeling. At the same time, integrating machine learning and data science can accelerate compound discovery, support sustainable synthesis, and predict clinical outcomes.

Achieving these goals will require interdisciplinary collaboration, equitable access to funding, and responsible stewardship of natural resources. We are reminded that some of the most transformative and lucrative therapies today, such as paclitaxel (from *Taxus brevifolia*, *Pacific yew* tree), morphine (from *Papaver somniferous*, the *Opium poppy*), and artemisinin (from *Artemisia annua*, sweet wormwood), originated from persistence in investigating plant-based compounds—often against conventional expectations or in the face of skepticism—and today save millions of lives.

The contributions in this Special Issue reaffirm that same spirit of perseverance in reporting the untapped potential of plant-derived bioactive compounds in addressing pressing health challenges.
